# Theodor Billroth: The Pioneer Gastrectomy Surgeon and His Contributions to the Evolution of General Surgery

**DOI:** 10.7759/cureus.68861

**Published:** 2024-09-07

**Authors:** Agostino Fernicola, Armando Calogero, Michele Santangelo

**Affiliations:** 1 Division of Endoscopic Surgery, Department of Clinical Medicine and Surgery, Azienda Ospedaliera Universitaria Federico II, Naples, ITA; 2 Division of Emergency Surgery, Department of Advanced Biomedical Sciences, Azienda Ospedaliera Universitaria Federico II, Naples, ITA

**Keywords:** billroth, billroth 2, billroth i reconstruction, biographies, gastrectomy, general surgery, historical vignette, historical vignettes, medical innovation, medical stories

## Abstract

Christian Albert Theodor Billroth, born in Rügen on April 26, 1829, is considered a pioneer of gastrectomy. Billroth entered the history of general surgery with his two famous methods of gastric resection. In his time, the diagnosis of stomach cancer was often extremely late because it was based exclusively on anamnesis and palpation and X-rays had not yet been discovered. This review aims to describe the history of a master of surgery such as Billroth, highlighting his attempts to develop gastrectomy techniques for the first time, which then influenced modern ones.

## Introduction and background

Unlike English and Scottish doctors, who often come from modest families, German and Austrian doctors come from wealthy families [1]. However, among the latter, Christian Albert Theodor Billroth (Rügen, April 26, 1829) was an exception [2]. In fact, his father was a Protestant pastor and died of tuberculosis when Billroth was five years old, leaving his wife and children in precarious economic condition [3]. Although his true passion was music, he enrolled at the Faculty of Medicine in Greifswald at the age of 19 and graduated in 1852 with the thesis *De natura et causa pulmonum affectionis quae nervo utroque vago dissecto exoritur*, in Berlin. At 31, he became a professor at the University of Zurich [3,4]. It was the beginning of a brilliant career that led him to become one of the pioneers of gastric resection [3]. The aim of this review is to describe the historical context in which Billroth was able to perform gastrectomies on humans and how he contributed to the development of modern gastrectomies.

## Review

Early career

**Figure 1:**
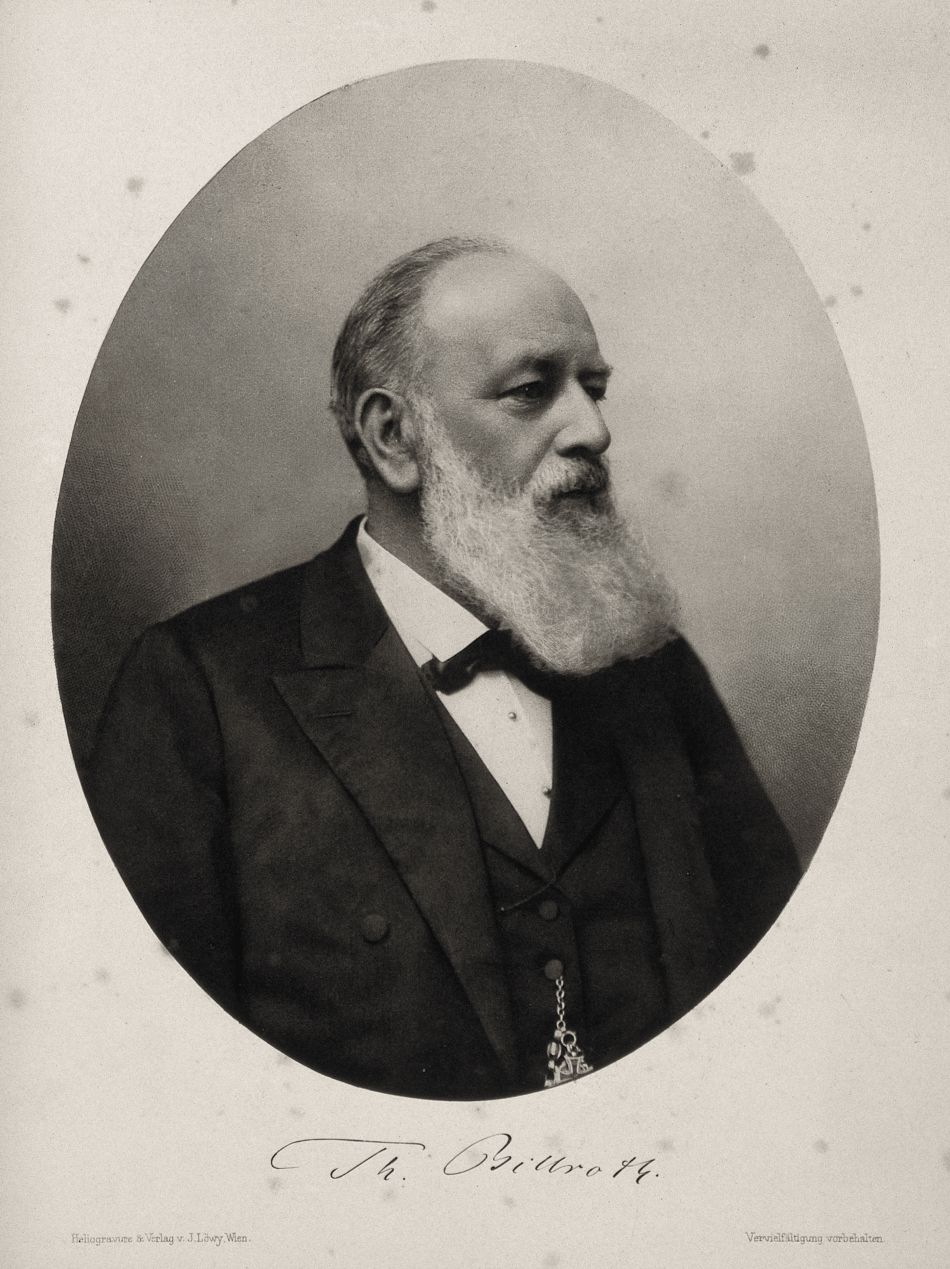
Christian Albert Theodor Billroth Image Credit: Wellcome Library, London This image is distributed under the terms of the Creative Commons Attribution-NonCommercial 4.0 International License.

At 38, the Austrian Emperor Franz Joseph called Billroth (Figure 1) to direct the II Surgical Clinic at the University of Vienna, to which Skoda, Rokitanski, and Hebra already belonged [4,5]. In short, Billroth became known in the academic world for his research: in fact, he described the red pulp of the spleen (*cords of Billroth*) and the tributary valves of the splenic vein (*cavernous veins of Billroth*) [1,3,5,6]. Finally, he gave his name to malignant lymphoma, known in those days as *Billroth's disease* [3]. By observing gastroenterostomies after pyloric resection was performed on dogs by Daniel Carl Theodor Merrem in 1810, Billroth demonstrated that he could perform esophagectomy on dogs in 1871 [3,4]. In 1873, he replicated the laryngectomy for the removal of a laryngeal tumor in a 36-year-old man, replacing the larynx with an artificial one, replicating the surgical technique in which Czerny had experimented on a dog [7-9]. In April 1879, J. Pean of Paris performed the resection of a pyloric cancer in a cachectic patient who later died on the fifth postoperative day [10]. In November 1880, L. Rydygier of Culm (Poland) performed a second gastrectomy, but his patient died only 12 hours after the operation [11,12]. At that time, there were several difficulties: the diagnosis of gastric cancer was late because it was based only on anamnesis and palpation of the patient's abdomen [6]. Furthermore, it took another 15 years before Röntgen discovered X-rays (1895) [13]. Two Billroth students (Winiwarter and Gussenbauer) reported that gastric juice did not digest suture material [14]. This discovery was of fundamental importance for the success of future gastrectomies [14].

Gastrectomies: Billroth I

Billroth waited until January 29, 1881, to decide to perform gastrectomy on a human being [3,6,15]. The patient was a 43-year-old woman, the mother of eight children, who, since October 1880, reported extreme weight loss, frequent threadlike pulses, palpable swelling in the epigastrium, and frequent nausea and vomiting [3,15,16]. The patient's name was Therese Heller, and the diagnosis according to Billroth was that of stenosing carcinoma of the pylorus [15,17]. After chloroform anesthesia, aided by Mikulicz, Billroth made a transverse incision corresponding to epigastric swelling, which appeared mobile and as large as an apple [1,3,6,17]. The diagnosis of carcinoma infiltrating the pylorus was confirmed, and it affected more than one-third of the distal portion of the stomach [3]. After ligating the vessels of the greater and lesser curvatures of the stomach, Billroth cut 30 cm upstream and downstream of the lesion and sutured the duodenal stump with carbolic silk around the residual gastric breach in continuity with the lesser curvature [3,18]. Therefore, the patient underwent end-to-end anastomosis between the duodenum and the residual stomach [3]. This operation is called *Billroth I* [3,18]. The operation lasted an hour and a half, including the induction of anesthesia [3]. At the end of the first postoperative week, the patient's condition was excellent, the wound had healed by primary intention, and Therese Heller had resumed eating semisolid foods [3]. However, the woman died four months after the operation due to metastases [3,18].

Gastrectomies: Billroth II

After this operation, Billroth's fear was that the resected stomach stoma was too large compared with that of the duodenum; therefore, he instructed his assistant, Dr. A. Wölfler, to study various surgical techniques for dogs [3-5]. Wölfler discovered a new gastroenterostomy technique in which the gastric lumen was reduced to the same diameter as the duodenal lumen and then the duodenal stump was sutured in continuity with the large gastric curvature (rather than with the small curvature) [1,2,4,6]. Billroth successfully applied this new surgical technique for gastrectomy (with anastomosis of the second jejunal loop with the residual stomach) in 32 patients and named it *Billroth II* [6,16]. On January 14, 1885, *Billroth II gastrectomy* was performed for the first time [6]. Owing to Billroth's surgical innovations, variants of his technique will subsequently be developed, which will be applied not only to gastric carcinoma but also to gastric ulcers and gastric stenosis [6,16]. Among the surgical techniques derived from Billroth's legacy, we mention esophagojejunal reconstruction on a Y-shaped loop according to Roux [4,17,19].

Later life

From an early age, Billroth was a lover of music, and in Zurich, he met Johannes Brahms, later becoming his friend [3,5]. In Zurich, he conducted a symphony orchestra [3]. Billroth died on February 6, 1894, in Opatija (Croatia), shortly after having just concluded a philosophical treatise on music entitled *Wer ist musikalisch?* [3-5]. The surgeon argued that musical talent was congenital, linked to the sense of rhythm, the ability to perceive the different pitches of timbre, and the intensity of sound [3-5].

The evolution of gastrectomy: from Billroth to modern times

The *Billroth I* and *Billroth II gastrectomy *surgical techniques have influenced the course of surgical history [20]. *Billroth I* *partial gastrectomy* involves the removal of the pylorus and gastroduodenal anastomosis [18]. At the time of Billroth, this surgical technique was indicated for distal gastric carcinoma, gastric ulcer, and gastric stenosis [20]. This technique allows to maintain the physiological path of food passage between the residual stomach and duodenum, compared to other anastomoses after partial gastrectomy for distal gastric carcinoma [20]. In the literature, many authors recommend also associating truncal vagotomy to reduce the risk of excessive gastric secretion in the residual gastric stump [16]. At the same time, the duodenal stump must be well vascularized and of sufficient length to reduce the risk of disinsertion of the papilla of Vater [20].* Billroth I gastrectomy* should be performed only in cases of small gastric lesions [18]. This concept makes it difficult to apply in cases of distal gastric cancer, in which a tumor-free margin of at least 5 cm must be obtained [17]. The gastroduodenal anastomosis can be performed manually or mechanically [20]. In the latter case, the length of the duodenal stump must be long enough to allow the entry of one of the two stapler tips [20]. Therefore, the* Billroth I technique* is not recommended even in the case of duodenal ulcers, in which a large portion of the duodenum must be removed, and in the case of an inflamed anatomical site of the future anastomosis [18,20]. As with the* Billroth II*, the *Billroth I technique* also has the disadvantage of bile influx into the residual gastric stump, with the subsequent risk of alkaline gastritis and bile reflux that negatively affect the patient's quality of life [18,20]. To overcome this disadvantage, over time, many surgeons have used the *Roux-Y *excluded intestinal loop reconstruction also after distal gastrectomy and not only for total gastrectomy [18]. This last intervention, in fact, distances the gastrojejunal anastomosis (in partial gastrectomy) or the esophagojejunal anastomosis (in total gastrectomy) from the point of entry of bile into the duodenum [20]. This is because the jejunal loop in continuity with the sutured duodenum is anastomosed with the alimentary loop at a distance of 40 cm from the first anastomosis [20]. In this way, the bile should go up this distance in an antiperistaltic direction to come into contact with the stomach (partial gastrectomy) or with the esophagus (total gastrectomy) [20]. *Roux-en-Y reconstruction* after distal gastrectomy is currently the most widely used surgical technique in cases of benign distal gastric cancer and early-stage malignant gastric cancer [17]. Likewise, this technique is the most easily performed in cases of total gastrectomy [17]. The anastomosis is infracolic (as in *Billroth II*) and isoperistaltic (unlike *Billroth II*) [20]. Thus, the cul-de-sac is located at the greater gastric curvature, unlike in the Billroth II where it is located at the lesser gastric curvature [20]. Partial gastrectomy with gastrojejunal anastomosis (*Billroth II*) is currently a valid alternative to *Roux-en-Y reconstruction* in cases of advanced-stage malignant gastric cancer [17-20]. The *Billroth II* technique involves anastomosis between the residual gastric stump and a jejunal loop of approximately 20-40 cm [19]. The anastomosis is infracolic and anisoperistaltic [20]. The *Polya variant* involves anastomosis with the entire length of the residual gastric division, while the *Finsterer variant* involves anastomosis with only a part of the gastric division [20]. Unlike the *Billroth I*, the *Billroth II* is associated with a lower risk of postoperative fistulas, probably due to the type of anastomosis (end-to-side in the *Billroth II *versus end-to-end in the *Billroth I*) and to the better vascularization [20].

The *Billroth I and Billroth II gastrectomy* can be characterized by postoperative sequelae, called *post-gastrectomy syndromes* (PSG): "dumping syndrome, alkaline reflux gastritis, chronic diarrheal syndrome with protein dispersion, afferent loop syndrome, and efferent loop syndrome" [17,20]. Over time, authors have tried to develop re-intervention techniques after the onset of PSG [20]. For example, the *Henley-Soupault-Bucaille method* (called TADE) involves reconversion from *Billroth II* to *Billroth I *[17,20]. Other techniques include partial degastrogastrectomy with Roux gastrojejunostomy and others the interposition of intestinal portions (colonic interposition or an isoperistaltic jejunal loop of 20-25 cm or an inverted antiperistaltic jejunal loop of 10 cm between the gastric and duodenal stumps) [20]. Over the years, the incidence of PSG has decreased due to the reduction in gastrectomy procedures (mostly performed only for gastric cancer or acute complications of gastric ulcers) [17-20]. However, PSG still occurs in about 20% of patients who have undergone gastrectomy [20]. *Billroth I *and *II *involve changes in the motor function of the stomach [18]. Resection with *Roux reconstruction* is the one that best prevents gastritis from alkaline reflux, compared to *Billroth I* and *II *[20]. However, *Roux reconstruction* can lead to gastric atony [20]. For this reason, some authors such as Mon and Cullen do not perform the section of the jejunal loop (as required by *Roux reconstruction*) but close the afferent jejunal loop with the residual gastric stump (techniques also called *"uncut" Roux*) [17,20]. Currently, PSG resolution techniques are classified into highly destructive and poorly destructive interventions [20]. Among the highly destructive ones, there are gastric resections with gastroenteroanastomosis and biliary diversion (according to Madura), reconversion according to the *Henley-Soupault-Bucaille technique* (TADE), and gastric stump resection with reconstruction according to Roux [17-20]. Among the poorly destructive ones, there are gastroenteroanastomosis with enteroenterostomy according to Braun and with the closure of the afferent loop and duodenum with stapler after Billroth I and the enteroenterostomy according to Braun with the closure of the afferent loop with stapler after Billroth II [17,20]. Randomized clinical trials are needed to compare major surgical procedures with the "uncut" Roux techniques in the future.

## Conclusions

Billroth was a pioneer surgeon of the first gastrectomies performed on humans. Despite the difficulties of an era in which diagnoses of gastric carcinoma were late or absent, he was the creator of the first partial gastrectomy with gastroduodenal anastomosis (Billroth I) and partial gastrectomy with gastrojejunal anastomosis (Billroth II). His surgical discoveries (still practiced today) were fundamental for the development of subsequent partial and total gastrectomies, such as the esophagojejunal reconstruction on a Y-shaped loop according to Roux. Finally, this pioneer of surgery is also remembered for his passion for music that never abandoned him throughout his life.
